# Elevated Lead, Nickel, and Bismuth Levels in the Peritoneal Fluid of a Peritoneal Endometriosis Patient without Toxic Habits or Occupational Exposure following a Vegetarian Diet

**DOI:** 10.3390/toxics11121009

**Published:** 2023-12-10

**Authors:** Andrea López-Botella, María José Gómez-Torres, Raquel Sánchez, José-Luis Todolí-Torró, Irene Velasco, Maribel Acién

**Affiliations:** 1 Service of Obstetrics and Gynecology, Unit of Human Reproduction, FISABIO—San Juan University Hospital, Carretera Alicante-Valencia s/n, 03550 Sant Joan d’Alacant, Alicante, Spain; andrea.lopez@ua.es (A.L.-B.); macien@umh.es (M.A.); 2 Biotechnology Department, Faculty of Sciences, University of Alicante, Carretera San Vicente del Raspeig s/n, 03690 San Vicente del Raspeig, Alicante, Spain; mjose.gomez@ua.es; 3 Department of Analytical Chemistry, Nutrition and Food Sciences, University of Alicante, Carretera San Vicente del Raspeig s/n, 03690 San Vicente del Raspeig, Alicante, Spain; r.sanchez@ua.es (R.S.); jose.todoli@ua.es (J.-L.T.-T.); 4 Gynecology Division, Faculty of Medicine, Miguel Hernández University, Carretera Alicante-Valencia s/n, 03202 Elche, Alicante, Spain

**Keywords:** ICP-MS/MS, potentially toxic elements, peritoneal fluid, gynecological pathologies, health effects, environmental contamination, endometriosis, peritoneal endometriosis

## Abstract

Potentially toxic elements (PTEs), found as environmental contaminants, have been related to endometriosis disease. In this context, the peritoneal fluid (PF) matrix has been poorly studied despite its importance. PF is the environment in which endometriotic lesions reside and communicate with surrounding tissues including tissues and nerve cells. In this work, our investigation group reports the special case of a peritoneal endometriosis patient presenting elevated lead, nickel, and bismuth levels in PF. This patient reported following a vegetarian diet and no toxic habits or occupational exposure. In conclusion, the elevated levels of PTEs found may result from a vegetarian diet or an unidentified environmental exposure source. This report provides new insights regarding the possible etiology of endometriosis disease and potential biomarkers for its diagnosis in early stages, although additional research is needed.

## 1. Introduction

Recent scientific works have established a connection between environmental contaminants and human reproductive health concerns [1]. While the extent of environmental exposure’s contribution to infertility remains uncertain, substantial evidence suggests its potential negative impact on fertility [2]. On this basis, environmental contaminants as heavy metals, which are also known as potentially toxic elements (PTEs) [3], have been identified as reproductive toxicants that adversely affect female fertility [4]. Despite the fact that a scarce number of studies are available, illnesses such as breast cancer, endometrial cancer, menstrual disorders, spontaneous abortions, pre-term deliveries, and stillbirths or endometriosis have been related to PTEs exposure [5]. Recently, the possible mechanisms on how heavy metals impact female fertility have been reviewed by Dring et al. [6]. Their research highlighted that exposure to selected toxic elements is associated with impaired oocyte maturation, ovulation, and fertilization. Thus, it might even impair further fetal development. Further to this, certain PTEs such as Cd and Pb have been correlated with impaired male fertility [7] due to an impairment of the endocrine function and gametogenesis or excess production of reactive oxygen species (ROS) [8,9].

On the other hand, certain PTEs (Cd, Pb, and Hg) are classified as endocrine-disrupting compounds (EDCs) [7]. EDCs are chemical pollutants that interact with the function of endogenous hormones, disrupting metabolic pathways [8]. For instance, cadmium (Cd) activates the estrogen receptor alpha (Erα), inducing an estrogenic activity [1,10], and has been related to the development of estrogen-dependent diseases [1]. Nevertheless, Cd has been associated with multiple adverse women’s reproductive health outcomes but the lack of evidence indicates the necessity for further research [11]. Analytical data examining the accumulation of PTEs in organs, tissues, and/or biological fluids could provide useful information about their correlation with the quality of the environment, lifestyle, and diet.

Peritoneal fluid (PF) is a normal sterile lubricating fluid found in the pelvic cavity that reduces the friction between the organs and the abdominal wall. It is present in a small and variable volume (2–10 mL) in a healthy person. In women, PF constitutes an extracellular fluid, representing plasma ultrafiltration [12] and ovarian exudation, and is caused by an increased permeability; it is reabsorbed by the mesothelial cells of the peritoneal cavity. However, ovary-related diseases such as endometriosis, among others, should be considered when women exhibit an excess of PF [13]. Furthermore, this fluid contains specific biomarkers of gynecological diseases [14] that could be useful for the diagnosis and prognosis of gynecological pathologies. In this sense, endometriosis is one of the most frequent benign gynecological diseases, affecting about 7–10% of women worldwide [15]. This painful disease arises from the growth of endometrial glands and stroma outside the uterine cavity [16]. Endometriosis has a major social impact as no reliable diagnostic tests exist; therapies are only symptomatic and fertility problems are related to the disease. While its pathogenesis is not fully elucidated, retrograde menstruation and coelomic metaplasia are the most widely accepted hypotheses [16]. Endometriosis seems to be a multifactorial disease but little is known about its etiology and growing evidence suggests that EDCs may be related to the disease [17]. Some elements such as Cd, lead (Pb), and mercury (Hg) are PTEs and EDCs whose relationship with endometriosis has been investigated [18,19].

In this case report, the multielemental profile of the PF fluid of a peritoneal endometriosis patient is described and compared with an age-matched healthy control patient. Lifestyle habits and expositional data of the patient are examined and discussed with information from the literature about how PTEs could be related to endometriosis disease.

## 2. Case Report

A 22-year-old woman was referred to the Service of Obstetrics and Gynecology at San Juan University Hospital, Alicante, Spain. Trans-vaginal ultrasonography (TVS) revealed a left ovarian cyst of 7.5 cm, which was diagnosed as serous cystadenoma. After the laparoscopic surgery, the patient was diagnosed with peritoneal endometriosis.

The patient did not have any comorbidities and was not taking any medications. Further to this, she had no smoking or drinking alcohol habits and lived in a small town where no environmental exposition was documented. Also, the patient was not occupationally exposed to PTEs.

PF samples were collected during the laparoscopic surgery in the operating room. The multielemental profile of the PF samples was explored to investigate the PTEs content in the samples.

## 3. Material and Methods

### 3.1. Lifestyle Habits Questionnaire

A habit survey was conducted. This questionnaire included clinical data (age, height, and weight), lifestyle habits related to nutritional information, toxic habits (smoking status, drug consumption, and alcohol intake), physical exercise, sociodemographic information, occupational status, occupational exposure, and, finally, data including environmental exposure. Study data were collected and managed using the REDCap (Research electronic data capture) tools hosted at Generalitat Valenciana-Fundación para el Fomento de la Investigación Sanitaria y Biomédica de la Comunitat Valenciana (GVA-FISABIO) [20,21]. REDCap is a secure web-based software platform specifically designed to support data collection for research studies, providing (1) an intuitive interface for validated data collection; (2) audit trails for tracking data manipulation and export procedures; (3) automated export procedures for seamless data downloads to common statistical software; and (4) procedures for data integration and interoperability with external sources.

### 3.2. PF Sample Collection

Peritoneal fluid samples were obtained by laparoscopy and collected by aspiration into sterile syringes. Then, the samples were filtered through a 20 µm filter to remove fibrin and cell aggregates and transferred to a glass centrifuge tube. After centrifugation (600× *g*, 10 min), the resulting supernatants were stored in the dark at −20 °C until their analysis. Informed consent was obtained from each patient after they received a comprehensive explanation of the objectives of the study. Ethical approval was granted by the Institutional Review Board of the San Juan University Hospital, Alicante, Spain (Committee protocol code: 19/344, date of approval: 17 December 2019).

### 3.3. PF Samples Analysis

The multielemental composition of the PF of the case was investigated using an Agilent 8900 ICP-MS/MS (Agilent Technologies, Santa Clara, CA, USA) equipped with an octopole reaction system (ORS) and axial acceleration technology. This method was previously validated by our research group [22]. PF samples were diluted 1:4 with ultrapure water according to the method developed by our investigation group [22].

## 4. Results

### 4.1. Results from the Habit Survey

The habits survey revealed that the patient followed a lacto-ovo vegetarian diet, consumed ecological food, and rarely or never ate canned food. No toxic habits were documented and no occupational or environmental exposure was reported, as mentioned above (Table 1).

### 4.2. Clinical Data and Surgery Findings

During the surgical procedure, several additional findings were made and described as follows: right ovary with an endometriosis implant and normal right tube; endometriosis vesicles in Douglas; and retroverted uterus with fibrous band on the right uterosacral ligaments with significant retraction and marked right ovarian fossa which endometriosis implants settle. Whitish implants were also observed on the parietal peritoneum of the Retzius space. Clinical data of the case patient can be found in Table 1. An age matched control was used to compare the analytes levels found in the PF extracted during surgery. The control patient had been diagnosed with a serous cystadenoma after a trans-vaginal ultrasonography (TVS) confirmed a right ovarian cyst of 8.6 cm. The control case did not have any comorbidities. She had no smoking or drinking alcohol habits and was taking no medications. She lived in a small town with no environmental exposition. Also, the control patient was neither occupationally exposed to PTEs. Clinical data of the control patient are available in Table 2.

### 4.3. Results of Pathological Tests and Toxicology Profile of PF

To compare the levels of analytes in the PF, the PF from an age-matched control patient was analyzed. Firstly, it was checked that the matrix was similar regarding the protein content using the signal of phosphorous and sulfur which gave us a ratio of 0.93 and 0.7, respectively. Also, argon signal was used to clarify that the sample matrix was similar, with a 0.99 ratio. Thus, proving that PF analytes content could be compared. Further to this, 10 PF samples from non-endometriosis patients were analyzed and included in this report to compare the analyte levels to use this data as reference. This group included women diagnosed with non-hormonally dependent benign ovarian cysts in which there may also be an underlying inflammatory process. Table S1 in the Supplementary Material summarizes demographic, biochemical, and expositional data regarding this group.

In contrast, when trace elements were under study, some of them showed different rations depending on the patient. Table 3 summarizes the concentrations found for 11 different trace elements and the two patients of interest. The third column contains the concentrations found for the control group of reference (n:10). The results revealed elevated PF barium (Ba), bismuth (Bi), cobalt (Co), nickel (Ni), and lead (Pb) ratios in the case patient compared to age-matched control: 4:1, 1.5:1, 5:1, 4:1, and 90:1, respectively (Table 3).

## 5. Discussion

### 5.1. Essential Human Elements

Co and Ni are essential trace elements but they are also classified as d PTEs [3]. Although the mean values for Co in biological fluids are typically low (0.16 µg/L in serum and 0.40 µg/L in urine) [23], we found that this patient had 1.39 ± 0.06 µg/L in PF, with a ratio of 5:1 compared to the healthy control. Co was found in concentrations below the LOD in the reference control group (Table 3). Co is an essential element that composes hydroxycobalamin (vitamin B12), which is essential for red blood cell production. Human dietary intake of cobalt varies between 5 and 50 μg per day, with inorganic Co being the most ingested form. Co is rapidly excreted in urine as it does not accumulate in the organism. Both anthropogenic and natural sources equally contribute to its emission into the atmosphere. The first one is due to the burning of fossil fuels and sewage sludge, phosphate fertilizers, mining, and industries that produce or process cobalt [24].

In the case of Ni, a significantly elevated level in the PF of the endometriosis patient was observed (40.4 ± 0.7 µg/L), with a 4:1 ratio compared to the control. Regarding the reference group, Ni was found in PF in concentrations of 1.9 to 5.1 µg/L. Although Ni serves as a cofactor for some metalloenzymes, no specific biological function has been identified for it in humans [25]. Previous studies have reported Ni levels in the serum and follicular fluid (FF) of women undergoing in vitro fertilization, with levels of 2.8  ±  2.4 and 3.2  ±  2.7 µg/L, respectively [26]. Environmental exposure to Ni could come from fossil fuel combustion and Ni-related industries. Also, Ni could be ingested through the consumption of plant and animal products. Vegetables such as spinach, legumes, and nuts contain high amounts compared to other foods. Other products such as baking powder and cocoa powder also contain this element as a possible result of the manufacturing process. Ni is also present in tobacco leaves due to metal accumulation in the soil. Concerning its absorption from food and water, it occurs by intestinal absorption although the absorption by skin contact can also take place [24]. In this case, the elevated Ni levels in the PF of the endometriosis patient might be attributed to her vegetarian diet as some studies reported that ovo-lacto-vegetarians eat higher amounts of Ni than people following a mixed diet [27].

### 5.2. Non-Essential Human Elements

Related to endometriosis and the exposition to PTEs, Cd and Pb are also considered EDCs, as previously mentioned. Regarding Pb, the results show high concentrations of Pb in the PF of the patient with peritoneal endometriosis, with a concentration of 75 ± 4 µg/L. In addition, the ratio for Pb was 90:1 compared to the control patient. In the reference group of women with no endometriosis, Pb was found in PF samples in a range of 1.9 to 4.8 µg/L, which are quite low concentrations compared to levels found in the case report. A recent study highlighted a synergistic effect of co-exposure to Pb and Cd on endometriosis [19]. Furthermore, PF Pb concentration was higher than the mean concentrations found in blood: 13.37 µg/L [28], and 19.61 µg/L [29]. While some studies have reported no association between Pb blood levels and endometriosis [30], others found higher concentrations in endometriosis patients (19.6 µg/L) compared to a control group (15.0 µg/L) [29]. A study performed among infertile Asian women, with and without endometriosis, found that Pb blood levels were associated with increased odds of endometriosis [28]. Nevertheless, another study found that Pb was at lower levels in the blood of women suffering endometriotic diseases compared to control patients [31]. Major sources of Pb exposure include gasoline, industrial emissions, and lead-based paint. However, in industrialized and affluent countries, exposure to Pb from these sources has significantly decreased. It is worth noting that near lead-emitting industries, inhalation, and ingestion of lead have increased and that cigarette smoking exacerbates Pb exposure. Pb is also ingested through diet. The amount of Pb ingested from diet is similar in a standard, vegetarian, vegan, and carnivore diet, which ranges from 0.033 mg per person per day in a vegetarian to 0.036 mg per person per day in a vegan diet [32]. In hair, it was found that greater values of lead in people following a vegetarian diet, which was thought to be probably due either to contaminated food or environmental factors [33]. Major dietary sources of lead include fruits, vegetables, plant products, starchy roots, oilseeds, legumes, and nuts [34] together with cereals, bakery products, and beverages. Also, Pb is used to lead-glaze pottery and is used for the storage of fruit juice. Finally, lead-soldered tin cans are used to contribute to Pb intake but nowadays are uncommon [24]. General environmental exposure together with the vegetarian diet that the endometriosis patient follows could explain the lead levels found in the PF of this patient.

In our study, Cd was not detected in the PF of the endometriosis patient. Cd blood levels have been associated with significantly increased odds of endometriosis [29,30] but conflicting results have been reported in different studies [11,31,35]. However, despite in vitro and in vivo evidence suggesting that Cd is a metalloestrogen, results from human studies evaluating the association between Cd and estrogen-dependent diseases in women provided conflicting results. So, it could be considered a risk factor for estrogen-dependent gynecological diseases [36].

Barium (Ba), despite being classified as a PTE, has not been associated with endometriosis. Our results show the presence of Ba in a concentration of 9.6 ± 0.8 µg/L in the PF of the endometriosis patient, with a signal ratio of 4:1 compared to the control. In the reference group, Ba was found in the range of 3 to 18 µg/L. The estimated daily Ba intake from food, water, and air is estimated to range from about 0.7 to 1.9 mg per day, with food being the primary source of intake for those who are not occupationally exposed [37]. Despite relatively high concentrations of Ba in soil, a limited amount is taken by plants, and lower concentrations get the human species. Pecan nuts and dry cocoa have notably high Ba levels (6.7 and 12 mg per kg, respectively) [24]. For its part, Bi and Ti are considered non-essential human elements but not PTEs [3]. In our results, Bi was found at 33.3 ± 1.6 µg/L concentrations in the PF of the case patient, representing a 1.5:1 ratio compared to the healthy control. In the reference group, this element was found in the range of 0.3 to 0.8 µg/L. Long-term exposure to Bi could result in toxicity in humans; however, it depends on the amount and nature of Bi species [38]. For the general population, the daily intake of food and water ranges from 5 to 20 µg. However, the specific concentrations for food items were not described. Moreover, Bi has pharmaceutical and cosmetic applications and thus constitutes a prolonged source of exposure for specific groups [24]. Cosmetic products often contain metallic powders, such as bismuth oxychloride, which is used to create a pearled shine effect [39]. Nevertheless, no direct relationship with endometriosis disease or reproductive health has been established.

Ti exposure has been associated with various adverse health effects but its specific impact on gynecological and reproductive health remains limited. In our study, we found that Ti was present at 60 ± 2 µg/L. When compared with the control, the ratio found for Ti was 1:1, which reflects similar levels for this analyte in both PF samples. Further to this, Ti was found in the reference group in concentrations from 12 to 219 µg/L. These results reflect variability in Ti exposure and consequently explain the concentration range found in the PF. The primary source of Ti exposure is the ingestion of TiO2 nanoparticles (also known as the additive E 171), which have been widely used in the food industry as a colorant in processed food. This additive is also present in toothpaste and medicines as an excipient in capsules or in film coating tablets [40]. However, due to their genotoxicity, this additive was forbidden by the European Union in February of 2022 (EU REGULATION 2022/63) [41]. As for the amount of Ti ingested through diet, an adult weighing 50 kg may consume approximately 10–35 mg of TiO_2_ [42]. A recent study found significantly higher Ti levels in healthy controls (179 µg/L) compared to women with diminished ovarian reserve (150 µg/L) when Ti was measured in FF [43].

As has been previously mentioned, Ni and Pb are PTEs found in cigarettes [44]. However, our endometriosis patient had no history of smoking. Also, we did not assess any occupational exposure in her current role as a digital artist. Her diet was within normality, without any notable habit that could explain the levels found. However, her vegetarian diet could explain slightly higher PTE levels in PF as Ni. Furthermore, no environmental exposure was recorded but an unknown source of environmental exposure could explain the PTEs found in PF.

## 6. Conclusions

The multielemental profile of the peritoneal fluid belonging to a peritoneal endometriosis patient revealed elevated concentrations of Pb (75 ± 4 µg/L), Ti (60 ± 2 µg/L), Ni (40.4 ± 0.7 µg/L), and Bi (33.3 ± 1.6 µg/L). Upon comparison with an age-matched control, we identified increased ratios for Pb (90:1), Ni (4:1), Ba (4:1), Co (5:1), and Bi (1.5:1). No toxic habits, occupational exposure, or environmental exposure were reported, which suggests that these elevated levels may result from a vegetarian diet or an unidentified environmental exposure source. Nevertheless, it is essential to acknowledge a primary limitation in this case report—the obtained results, while informative, fall short of providing conclusive evidence. This study serves as a preliminary examination and ongoing research with a more extensive study group is actively underway in our laboratories. We anticipate that the forthcoming publication of the results from this expanded study will furnish more definitive insights into the correlation between essential and non-essential elements, including potentially toxic elements (PTEs), and the manifestation of endometriosis.

## Figures and Tables

**Table 1:** Extracted data from the habits survey.

Item	Answer
Prematurity	No
Did your mother smoke during pregnancy?	No
Did your mother work during pregnancy?	No
Diet	
Ecological food consumption	Yes
Eggs consumption (4 times per week)	Yes
Meat consumption (2–3 times per week)	No
Cheese consumption (3 times per week)	Yes
Dairy milk consumption (1–2 glasses per day)	No
Fish consumption	No
Canned food consumption	Rarely or never
Orange consumption (2–3 times per week)	Yes
Tomato consumption (3 times per week or more)	Yes
Daily consumption of vegetable oils (walnuts, sesame, soy...)	No
Olive oil consumption (3 times per week or more)	Yes
Hazelnuts, walnuts, almonds consumption (1 time per week or more)	Yes
Alcoholic beverages consumption	No
Lifestyle habits	
Smoker	No
Passive smoker	No
Ex-smoker	No
Drugs consumption	No
Physical exercise	No
Occupational exposure	
Educational level	University
Profession	Digital artist
Occupational contact with substances (pesticides, pesticides, chemical substances, disinfectants, paints, foundries, industrial products, or other types of substances)	No
Environmental exposure	
Residence area	Rural
Nearby chemical industry that works with toxic products	No
Environmental contact with substances (pesticides, pesticides, chemical substances, disinfectants, paints, foundries, industrial products, or other types of substances)	No

**Table 2:** Clinical data of the case and control patients.

ID	Age (Years)	Height (cm)	Weight (kg)	BMI	Diagnosis	TSH mUI/L	CA125 (U/mL)
Case	22	166	52	18.9	Peritoneal endometriosis + Serous cystoadenoma	1.0	42.6
Control	19	158	56	20.0	Serous cystoadenoma	-	8

**Table 3:** Trace and ultra-trace metal elements and PTMEs were found in the peritoneal fluid samples. Samples were diluted 1:4 with ultrapure water. The measurements were performed by ICP-MS/MS. Concentrations are expressed in µg/L.

Analyte	Case Concentration (Mean ± SE)	Age-Matched Control Concentration (Mean ± SE)	Case/Age-Matched Control Ratio	Reference Control Group (Min–Max) Concentration
Ba	9.6 ± 0.8	<LOQ (2)	4:1	3–18
Bi	33.3 ± 1.6	<LOQ (0.5)	1.5:1	0.3–0.8
Co	1.39 ± 0.06	<LOD	5:1	<LOD
Mn	1.05 ± 0.02	3.78 ± 0.02	0.99:1	1.1–7.4
Mo	<LOQ (2)	3.2 ± 0.004	0.62:1	<LOD
Ni	40.4 ± 0.7	<LOD	4:1	2.9–5.1
Pb	75 ± 4	0.72 ± 0.00019	90:1	1.9–4.8
Rb	148 ± 6	135 ± 7	1.06:1	77–151
Sn	<LOD	<LOD	-	<LOD
Sr	50.0 ± 2	40.1 ± 0.6	1.26:1	16–54
Ti	60 ± 2	37 ± 0.07	0.92:1	12–219

Ba: Barium, Bi: Bismuth, Co: Cobalt, Mn: Manganese, Mo: Molybdenum, Ni: Nickel, Pb: Lead, Rb: Rubidium, Sn: Tin, Sr: Strontium, Ti: Titanium, <LOD: Below the Limit of Detection, <LOQ: Below the Limit of Quantification.

## Data Availability

The data presented in this study are available in Supplementary Materials.

## Supplementary Materials

The following supporting information can be downloaded at https://www.mdpi.com/article/10.3390/toxics11121009/s1. Table S1: Demographic, biochemical, and expositional data from the reference control group.
