# Supplementary material for: Elevated Lead, Nickel, and Bismuth Levels in the Peritoneal Fluid of a Peritoneal Endometriosis Patient without Toxic Habits or Occupational Exposure following a Vegetarian Diet
Source: Toxics. 2023 Dec 10;11(12):1009. doi: 10.3390/toxics11121009 (PMC10747131; doi:10.3390/toxics11121009)
Supplement: Supplementary file 1 [file toxics-11-01009-s001.zip › toxics-2709827-supplementary.pdf]

Table S1. Demographic, biochemical, and expositional data from the reference control group.

Parameter	Reference control group
Demographic data	
Age (min-max)	13-43
BMI (min-max)	17-25
Sterile (%)	10
Biochemical data	
TSH (mUI/L) (min-max)	1.25-2.71
CA125 (U/mL) (min-max)	2.60-24.6
Expositional status	
Current smoker (%)	10
Ex-smoker (%)	20
Occupationally exposed (%)	20
Environmentally exposed (%)	0
